# The taxonomic relevance of flower colour for *Epimedium* (Berberidaceae), with morphological and nomenclatural notes for five species from China

**DOI:** 10.3897/phytokeys.118.30268

**Published:** 2019-02-27

**Authors:** Yanqin Xu, Linjian Liu, Shaoxiong Liu, Yiming He, Renqing Li, Fei Ge

**Affiliations:** 1 College of Pharmacy, Jiangxi University of Traditional Chinese Medicine, Nanchang 330004, China Jiangxi University of Traditional Chinese Medicine Nanchang China

**Keywords:** intraspecific, morphology, perianth, polymorphism, population, Ranunculales

## Abstract

Morphological variations, particularly flower colour, could be considered as an evolutionarily and ornamentally significant taxonomic criterion for *Epimedium*. Our extensive field investigation based on population studies revealed abundant intraspecific variations in flower colour. Five species, (i.e., *E.acuminatum* Franch., *E.leptorrhizum* Stearn, *E.pauciflorum* K.C.Yen, *E.mikinorii* Stearn, and *E.glandulosopilosum* H.R.Liang) were found to possess polymorphic flower colour, which is first described and illustrated here. Moreover, all these species were found to be polymorphic in other diagnostic characters, such as the type of rhizome, the number and arrangement of stem-leaves, and/or their indumentum, which have not been adequately described in previous studies. Therefore, their morphological descriptions have been complemented and/or revised. We also provide notes on the morphology and nomenclature for each species. Additionally, a key to the species in China has been provided. The present study could serve as a basis for understanding their taxonomy and helping their utilisation as an ornamental plant.

## Introduction

*Epimedium* L. is the largest herbaceous genus of Berberidaceae, comprising ca. 60 species distributed in the temperate mountain regions from eastern Asia to northwestern Africa, with enormous distributional gaps within these regions (Stearn 2002; Xu et al. 2014a; Zhang et al. 2015). China represents the diversity centre for the genus, with ca. 50 endemic species (except *E.koreanum* Nakai) (Stearn 2002; Ying 2002). *Epimedium* is an attractive genus, both horticulturally and botanically, and has received increased attention in past decades (Avent 2010; Ward 2004; Ying 1975, 2001, 2002; Stearn 2002; Zhang et al. 2011, 2014, 2015; Liu et al. 2016, 2017). Since the 1980’s, further botanical exploration and collections in China have dramatically increased the number of *Epimedium* species in this country (Ying 1975; Ying et al. 2011; Stearn 2002; Xu et al. 2014a). However, many *Epimedium* species have been described for China based on a single locality and/or the descriptions were based on limited specimens or collections (Stearn 1997; Zhang et al. 2011, 2014; Xu et al. 2014a). Due to the lack of additional field investigation and observation of morphological characters, the morphological variation amongst many *Epimedium* species is not yet well understood (Xu et al. 2014a, 2014b; Zhang et al. 2015; Liu et al. 2016, 2017). From 1990 to 1998, Stearn used a small collection of *Epimedium* to describe 16 species, which were based on a limited number of cultivated specimens (Stearn 1990, 1993a, 1993b, 1995, 1996, 1997, 1998), and thus, the morphological descriptions of some species were incomplete and/or inaccurate (Buck 2003; Xu et al. 2014a; Zhang et al. 2015; Liu et al. 2016). Our recent studies have focused on the standardisation of morphological descriptions in the genus (e.g., Liu et al 2016, 2017).

The divergence of floral traits is a striking phenomenon in flowering plants, which plays an important role in ecology, plant systematics and conservation (Vaidya et al. 2018). Specifically, flower colour has been demonstrated to be an important feature for identifying and classifying species (Ying 2001; Stearn 2002; Ying et al. 2011). *Epimedium* has the greatest range of flower colour than any other genus of Berberidaceae, varying from white (e.g., *E.latisepalum* Stearn) and yellow (e.g., *E.ecalcaratum* G.Y.Zhong, *E.fangii* Stearn, *E.flavum* Stearn, and *E.franchetii* Stearn) to pink (e.g., *E.leptorrhizum* Stearn), and to purple (e.g., *E.epsteinii* Stearn, *E.pseudowushanense* B.L.Guo, and *E.zhushanense* K.F.Wu & S.X.Qian) (Stearn 2002; Ying et al. 2011). The flower colour variation could be evolutionarily significant for *Epimedium*. However, compared to interspecific variation, intraspecific variation in flower colour is relatively uncommon (Matsumura et al. 2006). Usually, the colour of flowers is constant at the species level, but *E.grandiflorum* C.Morren and *E.acuminatum* Franch. are believed to be heterochromic (Stearn 2002; Ying et al. 2011). *Epimediumgrandiflorum* varies greatly in flower colour from white to pale yellow or light purple to reddish-purple to deep pink or violet (Stearn 2002). For *E.acuminatum*, intraspecific colour variation from white to yellow to pale violet or purple has been reported sporadically (Ying et al. 2011; Zhang et al. 2011, 2015). However, systematic investigations of flower colour variation are scarce for *Epimedium*, with the taxonomic significance or mechanism of the variation currently unknown. The flower colour diversity may be a signal of plant speciation, which also may be one of the reasons for the difficulty in assessing it for taxonomy (Bradshaw et al. 1995; Matsumura et al. 2006; Hopkins and Rausher 2011). Therefore, the study of the flower colour variation is of great significance to the taxonomy ranking of *Epimedium*, as well as for its evolution (Wang et al. 2017).

As part of our ongoing efforts to study the *Epimedium* diversity in China, we studied the relevance of flower colour variation for the taxonomy of *Epimedium* based on extensive field studies for five species of Chinese *Epimedium*, with additional cultivation and herbaria studies. Moreover, we present a comprehensive revision on their taxonomic description, including the variation on rhizome morphology, the number and arrangement of stem-leaves, and indumentum type. Additionally, we provide an identification key for the species of *Epimedium* recognised in China. The results provide abundant and important information for the taxonomy of *Epimedium*, and subsidies in its exploration and utilisation, for example, as ornamental plants.

## Materials and methods

In *Epimedium*, some diagnostic features, especially those of flowers and inflorescences, are obscured or not visible in the herbarium material. Moreover, the flowers are of frail texture and deciduous. Therefore, it is difficult to infer the exact colouration of *in vivo* flowers by examining dried specimens. Therefore, the vegetative as well as reproductive characters were examined mainly in their natural habitats, while the herbarium specimens were used as aids. Herbarium specimens were examined from the following herbaria: CDBI, E, GXMI, HGAS, HIB, IBK, IBSC, IMD, JXCM, K, KUN, N, NAS, P, PE, and SM (acronyms according to Thiers, continuously updated).

All field investigations and observations were conducted in full bloom from 2012 to 2017. To investigate the morphological variation, we carried out field surveys throughout the Chinese distribution range of *Epimedium*, especially in Sichuan, Guizhou, Guangxi, Hubei, Hunan, Anhui and Jiangxi provinces and Chongqing municipality. In each province or municipality, a survey, as wide and thorough as possible, was conducted. The species that exhibited variations in flower colour and other characters were then observed and sampled (Table 1). Herbarium specimens collected during our fieldwork were deposited in the herbarium of Jiangxi University of Traditional Chinese Medicine (**JXCM**). The ecological information of the relevant populations was recorded. With the aim of confirming the identifications and better understanding the difference between individuals, as well as populations, 20–30 individuals were collected from each population and posteriorly transplanted to the Jiangxi University of Traditional Chinese Medicine for further observations and studies. Quantitative measurements of rhizome diameter, the number of stem-leaves, height of the flowering stem, length of inflorescence, and the number of flowers were recorded for each specimen. Simultaneously, the following discrete morphological characters were also observed: type of rhizome; indumentum; arrangement of stem-leaves; inflorescence morphology; variation of flowers (i.e., shape, colour and proportion); and the colour of pollen *in vivo*. In addition, particular attention was paid to preparing herbarium specimens, deposited at JXCM herbarium, and photographing plants and also their floral parts in their natural habitat.

**Table 1.** Location and habitat characters of five *Epimedium* species.

**Species**	**Population code**	**Location (China)**	**Elevation (m)**	**Latitude (N)**	**Longitude (E)**
* E. acuminatum *	CQNC*	Tianxing, Nanchuan, Chongqing	899	29°03'N, 107°07'E	
	SCEM*	Emei Mountain, Leshan, Sichuan	1049	29°34'N, 103°25'E	
	SCMP*	Muping, Baoxing, Sichuan	1248	30°20'N, 102°48'E	
	SCSL*	Shangli, Yaan, Sichuan	862	30°11'N, 103°04'E	
	SCSS*	Shuangshi, Lushan, Sichuan	928	30°15'N, 102°55'E	
	SCYJ*	Siping, Yingjing, Sichuan	1448	29°43'N, 102°37'E	
	GZNL	Nanlong, Kaiyang, Guizhou	986	27°05'N, 107°05'E	
	GZYH	Hekan, Yanhe, Guizhou	637	29°02'N, 108°09'E	
	GZZA	Fengyi, Zheng’an, Guizhou	792	28°43'N, 107°51'E	
	CQFL	Mawu, Fuling, Chongqing	935	29°31'N, 107°19'E	
	CQYC	Chashanzhuhai, Yongchuan, Chongqing	762	29°28'N, 105°58'E	
	SCHJ	Fubao, Hejiang, Sichuan	723	29°28'N, 105°59'E	
	SCMB	Dazhubao, Mabian, Sichuan	1147	29°00'N, 103°30'E	
	SCXH	Xiaohe, Lushan, Sichuan	1192	30°29'N, 103°07'E	
	SCCM	Caomigang, Lushan, Sichuan	1534	30°26'N, 103°05'E	
	SCQL	Youzha, Qionglai, Sichuan	1067	30°26'N, 103°14'E	
	SCMT	Naobao, Qionglai, Sichuan	1468	30°25'N, 103°07'E	
	SCLG	Lingguan, Baoxing, Sichuan	1068	30°18'N, 102°48'E	
	SCWN	Wanniansi, Emei Mountain, Sichuan	1110	28°43'N, 107°51'E	
* E. leptorrhizum *	GZGY*	Shuikousi, Guiyang, Guizhou	1145	26°34'N, 106°44'E	
	GZST*	Ganlong, Songtao, Guizhou	907	28°19'N, 108°41'E	
	HNBJ*	Zhuping, Baojing, Hunan	978	28°36'N, 109°12'E	
	HBGP	Gongjiaping, Enshi, Hubei	1523	30°10'N, 109°44'E	
	HBMF	Mufu, Enshi, Hubei	1080	30°17'N, 108°56'E	
	HBLC	Fubaoshan, Lichuan, Hubei	1386	30°12'N, 108°43'E	
	HBTB	Tuanbao, Lichuan, Hubei	1200	30°22'N, 109°07'E	
	GZLC	Longchang, Kaili, Guizhou	796	26°39'N, 107°56'E	
	GZLS	Lingshan, Guiyang, Guizhou	1207	26°36'N, 106°42'E	
	GZMH	Muhuang, Yinjiang, Guizhou	1300	28°02'N, 108°42'E	
	ZJLQ	Cukeng, Longquan, Zhejiang	1210	27°55'N, 119°10'E	
	GDNL	Ruyuan, Shaoguan, Guangdong	670	24°55'N, 113°03'E	
* E. pauciflorum *	SCWC*	Yanmen, Wenchuan, Sichuan	1817	31°27'N, 103°34'E	
	SCMS*	Miansi, Wenchuan, Sichuan	1780	31°35'N, 103°49'E	
	SCJZ*	Jingzhou, Maoxian, Sichuan	1800	31°42'N, 103°53'E	
* E. mikinorii *	HBES*	Baiguo, Enshi, Hubei	754	30°14'N, 109°22'E	
	HBXT*	Xintang, Enshi, Hubei	1370	30°13'N, 109°41'E	
* E. glandulosopilosum *	CQWX*	Tongcheng, Wuxi, Chongqing	1161	31°23'N, 109°46'E	

*: the population with flower colour variation

The Flora of China (Ying et al. 2011) and the monograph of the genus *Epimedium* (Stearn 2002) were followed to describe the vegetative and reproductive characters of the studied species.

## Results

Epimedium*acuminatum* Franch., *E.leptorrhizum* Stearn, *E.pauciflorum* K.C.Yen, *E.mikinorii* Stearn and *E.glandulosopilosum* H.R.Liang, were found with intraspecific flower colour variation. Moreover, all these species were also morphologically variable in the number and arrangement of stem-leaves, the type of rhizomes and/or indumentum. Location, code, latitude, longitude, and elevation of the referred populations are presented in Table 1. The taxonomic descriptions, related illustrations, and information on the five species were updated below.

### 1. Epimedium acuminatum

Taxon classificationPlantaeRanunculalesBerberidaceae

Franch., Bull. Soc. Bot. France 33: 109. 1886

Fig. 1

Epimedium komarovii H.Léveillé, Fedde Repert. Spec. Nov. Regni Veg. 7: 259. 1909. Type: CHINA. Guizhou: Pin-Fa, 1908, *Cavalerie 954* (holotype: E00270388!).
Epimedium simplicifolium T.S.Ying, Acta Phytotaxon. Sin. 13: 51. 1975. Type: CHINA. Guizhou: Wuchuan, 9 May 1928, *P. C. Tsoong 606* (holotype: PE01432137!; isotype: PE01432138!).
Epimedium chlorandrum Stearn, Kew Bull. 52: 660. 1997. Type: CHINA. Sichuan: Baoxing, cultivated in England, Hampshire, Kilmeston, Blackthorn Nursery, April 1996, *Ogisu 94003* (holotype: K000340098!).

#### Type.

CHINA. Guizhou: 1858, *Perny* s.n. (holotype: P, barcode P02327614!; isolectotype: P, barcode P02327612!).

#### Description.

Herbs 20–80(–110) cm tall. Rhizome compact, sometimes long creeping, 2–8 mm in diam. Leaves basal and cauline, usually trifoliolate or occasionally unifoliolate; leaflets of trifoliolate leaves narrowly ovate or lanceolate, 3–19.8 × 1.5–8.9 cm, apex long acuminate, base cordate, lobes rounded or acute, those of the lateral leaflets very unequal; unifoliolate leaves ovate or broadly ovate, 8.7–20 × 6.8–11.5 cm, apex acuminate, base cordate, lobes equal, rounded or rarely acute; leaves leathery when mature, margin spinous-serrate, spines 1–2 mm long, adaxially deep green, glossy, abaxially glaucous, papillose, with dense or sparse shortly appressed stout bristle-like hairs and sometimes densely sericeous. Flowering stem with 2 trifoliolate opposite leaves, sometimes 3-whorled trifoliolate leaves or 2 opposite unifoliolate, rarely with 2 opposite leaves with one trifoliolate and the other unifoliolate, 3 whorled leaves with one trifoliolate and two unifoliolate or 3 whorled unifoliolate leaves. Panicle 6–70(–108)-flowered, 6–33 cm long, with lower peduncles loosely 2–5-flowered, glabrous or occasionally glandular-hairy. Pedicel 1–5 cm long. Flowers large, 3–5 cm in diam. Outer sepals 4, blunt, outer pair ovate-oblong, ca. 3 × 2 mm, inner pair broadly obovate, ca. 4.5 × 4 mm; inner sepals ovate-elliptic, 6–21 × 3–9 mm, apically acute, white, yellowish, pale rose or rose, petals pale yellow, yellow, pale violet, reddish-purple, purple-yellow, pale purple or purple. Petals curving outward, horn-shaped, much longer than inner sepals, 15–25 mm, tamping from the swollen but lamina-less base. Stamens 3–4 mm long; anthers yellow or green, ca. 2.5 mm long. Follicles oblong, ca. 20 mm long, style rostriform. Seeds numerous.

#### Distribution and habitat.

*Epimediumacuminatum* is one of the most widespread species in the genus, distributed in Sichuan, Guizhou, Chongqing, and northern Yunnan. Its large distribution area is predominantly characterised by mountain land. *Epimediumacuminatum* is often found on mountain slopes, forest edges or weedy slopes with elevations ranging from 270 m to 2400 m (Fig. 2).

#### Phenology.

*Epimediumacuminatum* flowers from April to June, and fruits from May to July.

#### Taxonomical remarks.

Before this study, flower colour variation in *E.acuminatum* had already been recognised. Overall, yellow (*B. Y. Peng 47073*, *F. T. Wang 23329*, *D. Y. Peng 47070*, *W. P. Fang 802*, *Anon. 86*, *Z. X. Qu 1305*, *Q. H. Chen et al. 9411*) or pale yellow (*Sanxia Exped. 0821*, *X. B. Zhang 19*), purple (*G. H. Yang 54343, Sichuan Econ. Pl. Exped. 0030*, *K. J. Guan et al. 0273*, *Xiong & Z. L. Zhou 91045*, *Z. Y. Liu 15500*, *T. H. Tu 3116*, *S. Z. He 96410*, *S. W. Teng 0008*, *P. Zhao 807*, *Y. Tsiang 4994*) or pale purple (*P. Zhao 762*, *Jinfoshan Exped. 0330*, *Jinfoshan Exped. 0202*, *G. F. Li 60324*), purple-whitish (*T. T. Yu 312*, *Z. Z. Guo 403*, *J. M. Yuan 003* and *J. M. Yuan 005*, *S. W. Tfng 90093*, *Z. S. Zhang et al. 401131*), and white (*K. J. Guan et al. 477, C. H. Li 97-301*, *K. Y. Lang 3002*, *K. J. Guan et al. 165*) or whitish (*Z. X. Qu 1057*, *Sanxia Exped. 0729* and *Sanxia Exped. 0909*) were the most frequently recorded flower colours. However, specific colours, for example, reddish (*S. P. Pong 6108*), yellowish purple (*X. Y. He 4050*; *T. C. Pan & G. F. Wu 105*), and pale purple-green (*Z. Y. Wu 60*) have also been examined from specimens. Colour differences among individuals of the same location have been slightly recorded, but both yellow and purple flowers were recorded in *Sichuan Econ. Pl. Exped. 0013*. Moreover, continuous variations from yellow to white and from yellow to pale reddish-purple were remarked by *T. H. Tu 2763* and *B. L. Guo 0608*, respectively.

Two synonyms are included in *E.acuminatum*, namely *E.simplicifolium* and *E.chlorandrum*. There were only two specimens of *E.simplicifolium* for reference. The holotype (*P. C. Tsoong 606*) recorded yellow flowers with purplish red petals. Since these descriptions were based on flowers that are not fully open – the outer sepals soon falling – the outermost is formed of inner sepals. Therefore, the “yellow flower” actually is “yellow or yellowish inner sepals”. Another specimen (*S. Z. He 96410*) recorded a purplish red flower, and the inner sepals spotted with purplish red. *Epimediumchlorandrum* has six specimens. Greenish inner sepals and pale yellow petals have been described in the holotype (*Ogisu 94003*). Pale yellow inner sepals and petals were recorded in *B. L. Guo 0540* and *B. L. Guo 0607* while *B. L. Guo 0606* described the colour variation of inner sepals as anything from pale yellow to pale purplish red. And *B. L. Guo 0608* also described both inner sepals and petals from pale yellow to pale purplish red.

**Figure 1.**
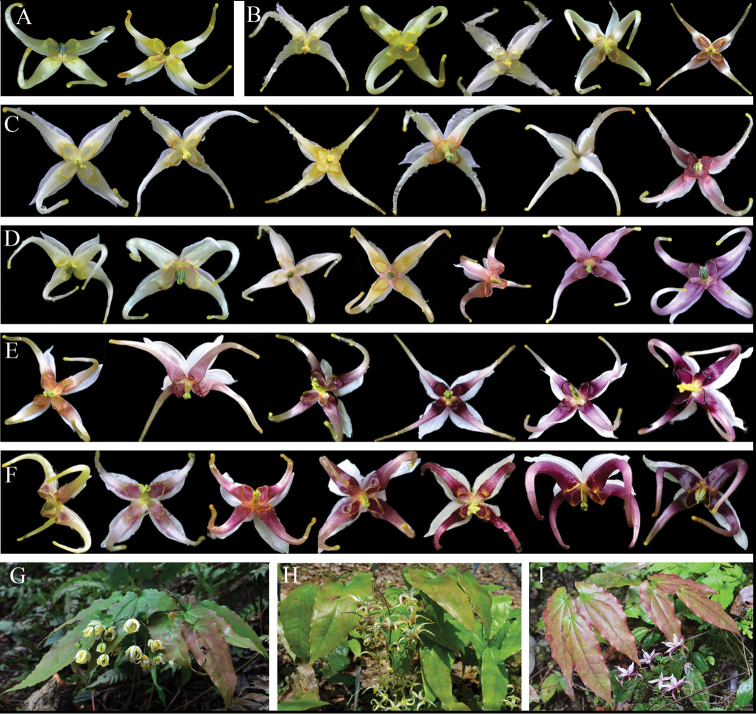
The flower colour variations of different populations of *E.acuminatum*. **A** SCMP, mainly yellow **B** SCSL, mainly yellow, occasionally purple-yellow at the base of petals **C** CQNC, mainly yellow, occasionally rose-purple **D** SCSS, from yellow to purple **E** SCEM, mainly purple, occasionally purple-yellow **F** SCYJ, mainly purple, occasionally purple-yellow **G** Individual with yellow flowers **H** Individual with purple-yellow flowers **I** Individual with purple flowers.

Based on a field survey at the population level, we observed more extensive and continuous colour variation from pale yellow to dark purple (Fig. 1). Pure white flowers have not been observed, but the pale yellow and whitish are very close to white. We therefore speculate that the white flowers described in the specimens might represent pale yellow or whitish flowers instead. Regarding the yellow and purple flowers, there was abundant colour variation among populations as well as among individuals. For example, yellow ranged from pale yellow to yellow, while purple ranged from pale violet or reddish-purple to purple and dark purple. Moreover, there were also many transitions between yellow and purple.

For *E.acuminatum*, the populations that showed uniform colour (yellow or purple) were excluded from the illustration in this study. We mainly focused on the populations that presented colour variation (Fig. 1). For example, SCMP showed mainly yellow flowers; only one out of the 30 individuals presented diverse colour, with a yellow spur tinged with a ray of rose inside the base of the petals. SCSL mainly had yellow flowers; parts of individual flowers showed pale yellow, and one individual presented purple-yellow at the base of the petals. CQNC primarily displayed yellow flowers; several individuals revealed a rose margin at the base of the petals, and one individual revealed reddish-purple flowers. SCSS was the most special population with 20 individuals had purple flowers while the rest (10 individuals) had yellow flowers. Moreover, SCSS was the only population for which the colour showed a continuous variation from yellow to purple. The flowers of SCEM and SCYJ were mainly with purple flowers, and purple-yellow occasionally (Fig. 1). In the above populations, SCMP and SCSS were *E.chlorandrum*.

**Figure 2.**
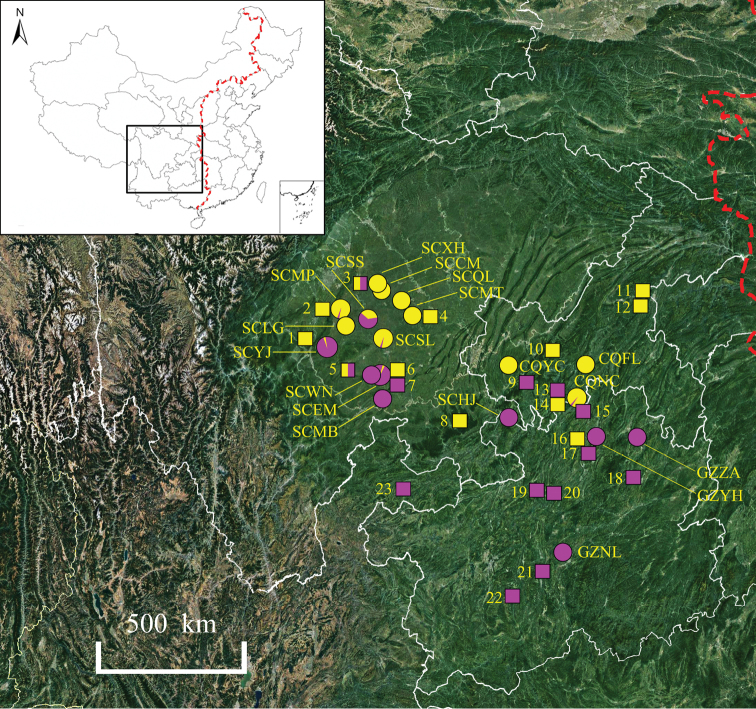
Geographical variation in flower colour patterns within *E.acuminatum*. The circles and boxes represent wild populations and herbarium specimens, respectively. The colour of the circles and boxes represent flower colour. The 19 population codes are shown in Table 1. The left area of the red dotted line is western China. The information of 23 representative herbarium specimens is as follow: 1. *B. Y. Peng 47070*; 2. *B. L. Guo 0540*; 3. *B. L. Guo 0608*; 4. *B. L. Guo 0607*; 5. *Sichuan Econ. Pl. Exped. 0013*; 6. *F. T. Wang 23329*; 7. *G. H. Yang 54343*; 8. *K. Y. Lang 3002*; 9. *Sichuan Econ. Pl. Exped. 0030*; 10. *T. C. Pan & G. F. Wu 105*; 11. *Sanxia Exped. 0729*; 12. *Sanxia Exped. 0821*; 13. *K. J. Guan et al. 0273*; 14. *X. B. Zhang 19*; 15. *Jinfoshan Exped. 0202*; 16. *Q. H. Chen et al. 9411*; 17. *J. M. Yuan 003; 18. Z. S. Zhang et al. 401131*; 19. *Y. Tsiang 4994*; 20. *S. Z. He 96410*; 21. *P. Zhao 807*; 22. *S. W. Teng 0008*; 23. *Z. Y. Wu 60*.

Combing the geographical distribution of specimens (both field and herbarium specimens) and their flower colour variation of *E.acuminatum*, we found an interesting result (Fig. 2). Geographic variation in flower colour pattern within *E.acuminatum* showed a north-south geographic trend. The specimens from the northern area of its distribution mainly have yellow flowers, while the southern ones usually have purple flowers. The polymorphism of flower colour is mainly concentrated in the northwest of its distribution area.

In addition, the number and arrangement of the stem-leaves were significantly diverse in *E.acuminatum*. It commonly showed 2 trifoliolate opposite leaves, sometimes 3-whorled trifoliolate leaves (*G. F Li 60514*, *Y. Tsiang 4994*) or 2 opposite unifoliolate (*S. Z. He 96410*, *P. C. Tsoong 606*), rarely with 2 opposite leaves with one trifoliolate and the other unifoliolate (*J. H. Xiong 30469*), 3 whorled leaves with one trifoliolate and two unifoliolate (*J. H. Xiong 30469*) or 3 whorled unifoliolate leaves.

#### Additional specimens examined.

CHINA. **Sichuan**: Chengxiang, Tianquan, 1100–1200 m, 24 March 1983, *B. Y. Peng 47070* and *B. Y. Peng 47073* (CDBI, yellow flower); Jiulaodong, Mt. Emei, 1800 m, 02 May 1957, *G. H. Yang 54343* (PE, KUN, HIB, NAS, purple flower); Lianhuashi, Mt. Emei, 1950 m, 11 May 1964, *K. J. Guan et al. 477* (PE, white flower); Mt. Emei, 1100 m, 11 June 1933, *S. P. Pong 6108* (PE, reddish flower); Mt. Emei, 1050 m, 13 March 1997, *C. H. Li 97*–*301* (PE, white flower); Mt. Emei, 900 m, 16 April 1932, *T. T. Yu 312* (N, purplish white flower); Mt. Emei, 2000 m, 15 July 1931, *F. T. Wang 23329* (PE, yellow flower); Mt. Emei, 900 m, 16 April, *T. T. Yu 312* (PE, purplish white flower); Mt. Emei, 1700 m, 27 May 1959, *Z. Z. Guo 403* (PE, purplish white flower); Mt. Emei, 1952, *J. H. Xiong 30469* (PE, 3 whorled leaves with one trifoliolate and two unifoliolate; IBSC, 2 opposite leaves with one trifoliolate and the other unifoliolate); Mt. Emei, 1049 m, 4 April 2015, *Y. Q Xu & S. X. Liu 2015029* (JXCM, mainly purple flower); Mt. Emei, 1110 m, 10 April 2017, *Y. M. He et al. 2017006* (JXCM, purple flower); Biexiandong, Jiang’an, 270 m, 5 April 1964, *K. Y. Lang 3002* (PE, white flower); Wuzhuang, Hongya, 1150 m, 20 April 1959, *Sichuan Econ. Pl. Exped. 0013* (SM, yellow and purple flower); Muping, Baoxing, 13 May 2005, *B. L. Guo 0540* (IMD, pale yellow flower); Dachuan, Lushan, 1484 m, 4 May 2006, *B. L. Guo 0606* (IMD, yellow flower); Youzha, Qionglai, 4 May 2006, *B. L. Guo 0607* (IMD, pale yellow flower); Dachuan, Lushan, 1484 m, 4 May 2006, *B. L. Guo 0608* (IMD, from yellow to pale reddish-purple); Shangli, Ya’an, 862 m, 7 April 2015, *Y. Q Xu & S. X. Liu 2015027* (JXCM, yellow flower); Siping, Yingjing, 1448 m, 8 April 2015, *Y. Q Xu & S. X. Liu 2015026* (JXCM, mainly purple flower); Fubao, Hejiang, 723 m, 10 April 2016, *S. X. Liu & J. X Zhu 2016007* (JXCM, purple flower); Dazhubao, Mabian, 1147 m, 13 April 2016, *S. X. Liu & J. X Zhu 2016008* (JXCM, purple flower); Dachuan, Lushan, 1192 m, 15 April 2016, *S. X. Liu & J. X Zhu 2016009* (JXCM, yellow flower); Dachuan, Lushan, 1534 m, 16 April 2016, *S. X. Liu & J. X Zhu 2016010* (JXCM, yellow flower);Youzha, Qionglai, 1067 m, 16 April 2016, *S. X. Liu & J. X Zhu 2016011* (JXCM, yellow flower); Nanbao, Qionglai, 1468 m, 16 April 2016, *S. X. Liu & J. X Zhu 2016012* (JXCM, yellow flower); Lingguan, Baoxing, 1068 m, 18 April 2016, *S. X. Liu & J. X Zhu 2016014* (JXCM, yellow flower); Shuangshi, Lushan, 928 m, 11 April 2015, Y. Q. Xu *& S. X. Liu 2015024* (JXCM, purple flower and yellow flower); Baoxing, cultivated at ENGLAND. Hampshire: Kilmeston, Blackthorn Nursery, April 1996, *Ogisu 94003* (holotype, K). **Chongqing**: Baiwuping, Nanchuan, 850 m, 16 April 1957, *G. F. Li 60514* (IBSC, KUN, NAS, 3-whorled trifoliolate leaves, reddish-purple flower); Tianxing, Nanchuan, 899 m, 27 March 2015, *Y. Q Xu & S. X. Liu 2015035* (JXCM, yellow flower); Nanchuan, 1500–1800 m, 16 May 1928, *W. P. Fang 802* (PE, IBSC, N, yellow flower); Xiaohe, Nanchuan, 1000 m, 10 May 1985, *Z. Luo 0033* (CDBI); Mt. Jinfo (Jinfoshan), Nanchuan, 1700–1900 m, 13 April 1964, *K. J. Guan et al. 0273* (CDBI, PE, purple flower); Mt. Jinfo (Jinfoshan), Nanchuan, 1550 m, 28 May 1957, *J. H. Xiong & Z. L. Zhou 91045* (KUN, purple flower); Mt. Jinfo (Jinfoshan), Nanchuan, 880 m, 25 May 1935, *X. Y. He 4050* (NAS, yellowish purple flower); Hetaoping, Mt. Jinfo (Jinfoshan), Nanchuan, 28 May 1935, *X. Y. He 4148* (NAS, whitish flower); Hetaoping, Mt. Jinfo (Jinfoshan), Nanchuan, 28 April 1935, *X. B. Zhang 19* (NAS, pale yellow flower); Mt. Jinfo (Jinfoshan), Nanchuan, 3 June 1935, *Anon. 86* (NAS, yellow flower); Yangyuping, Mt. Jinfo (Jinfoshan), Nanchuan, 1800 m, 14 May 1986, *Jinfoshan Exped. 0330* (PE, pale purple flower); Mt. Jinfo (Jinfoshan), Nanchuan, 720 m, 2 April 1996, *Z. Y. Liu 15500* (PE, purple flower); Mt. Jinfo (Jinfoshan), Nanchuan, 1200 m, 2 June 1935, *Z. X. Qu 1057* (PE, IBSC, whitish flower); Daheba, Mt. Jinfo (Jinfoshan), Nanchuan, 1020 m, 8 April 1964, *K. J. Guan et al. 165* (PE, white flower); Fenghuangsi, Nanchuan, 2050 m, 12 June 1935, *Z. X. Qu 1305* (PE, yellow flower); Delong, Nanchuan, 1500 m, 12 May 1986, *Jinfoshan Exped. 0202* (PE, pale purple flower); Nanchuan, 4 April 1933, *T. H. Tu 2763* (PE, yellow-white flower); Nanchuan, 1900 m, 22 May 1933, *T. H. Tu 3116* (PE, purple flower); Fangheba, Nanchuan, 550 m, 5 April 1957, *G. F. Li 60324* (PE, pale purple flower); Mt. Jinyun (Jinyunshan), Beibei, 700 m, 4 April 1963, *T. C. Pan & G. F. Wu 105* (PE, yellowish purple flower); Wangerbao Nature Reserve, Wanzhou, 977–1221 m, 25 April 2008, *Sanxia Exped. 0729* and *Sanxia Exped. 0909* (PE, whitish flower); Longju, Wanzhou, 981–1143 m, 27 April 2008, *Sanxia Exped. 0821* (PE, pale yellow flower); Simian, Jiangjin, 1200 m, 16 April 1959, *Sichuan Econ. Pl. Exped. 0030* (KUN, NAS, purple flower); Mawu, Fuling, 935 m, 7 April 2016, *S. X. Liu & J. X Zhu 2016005* (JXCM, yellow flower); Chashanzhuhai, Yongchuan, 762 m, 9 April 2016, *S. X. Liu & J. X Zhu 2016006* (JXCM, yellow flower). **Guizhou**: Fengyi, Zhengan, 700–1000 m, May 1992, *Q. H. Chen et al. 9411* (HGAS, yellow flower); Fengyi, Zhengan, 700 m, 1 June 1977, *J. M. Yuan 003* and *J. M. Yuan 005* (HGAS, purple-whitish flower); Huajiang, 1100 m, 1 April 1987, *P. Zhao 762* (HGAS, pale purple flower); Anshun, 25 May 1935, *S. W. Teng 0008* (IBSC, purple flower); Liuchongguan, Guiyang, 1300 m, 21 April 1987, *P. Zhao 807* (HGAS, purple flower); Maoshajing, Guiyang, 16 April 1936, *S. W. Tfng 90093* (IBSC, purple-whitish flower); Tongzi, 20 May 1930, *Y. Tsiang 4994* (PE, purple flower, 3-whorled trifoliolate leaves); Tuanlong, Yinjiang, 1090 m, 10 April 1964, *Z. S. Zhang et al. 401131* (PE, purple-whitish flower); Shuikousi, Guiyang, 1400 m, 16 March 1959, *Qiannan Exped. 42* (PE, KUN, reddish-purple flower); Suiyang, 1100 m, 10 April 1994, *S. Z. He 96410* (PE, 2 opposite unifoliolate leaves, purple flower); Longdong, Wuchuan, 9 May 1928, *P. C. Tsoong 606* (PE, KUN, IBSC, 2 opposite unifoliolate leaves); Nanlong, Kaiyang, 986 m, 28 March 2016, *S. X. Liu & J. X Zhu 2016002* (JXCM, purple flower); Hekan, Yanhe, 637 m, 1 April 2016, *S. X. Liu & J. X Zhu 2016003* (JXCM, purple flower); Fengyi, Zhengan, 792 m, 4 April 2016, *S. X. Liu & J. X Zhu 2016004* (JXCM, purple flower). **Yunnan**: Shuanghe, Weixin, 1480 m, 3 June 1960, *P. Di 1076* (KUN); Chaotianma, Yiliang, 1973, *Z. Y. Wu 60* (KUN, pale purple-green flower).

### 2. Epimedium leptorrhizum

Taxon classificationPlantaeRanunculalesBerberidaceae

Stearn, J. Bot. 71: 343. 1933

Fig. 3

Epimedium brachyrrhizum Stearn, Kew Bull. 52: 659. 1997. Type: CHINA. Guizhou: Mt. Fanjingshan, cultivated in the USA, Massachusetts, Hubbbardston, *Darrell Probst CPC940495* (holotype: K000340100!).

#### Type.

CHINA. Guizhou: Guiyang, *Bodinier 2184* (holotype: P, barcode P00568282!; isotypes: P, barcodes P00572792!, P00572793!, P00572800!, P00572801!, P00572802!).

#### Description.

Herbs 12–50 cm tall. Rhizome long creeping, or compact, 1–5(–8) mm in diam.; internodes sometimes to 20 cm. Leaves basal and cauline, trifoliolate or occasionally unifoliolate. Leaflets of trifoliolate leaves narrowly ovate or ovate, 3–16 × 2–8.6 cm, apex long acuminate, base deeply cordate with usually rounded lobes nearly touching, those of the lateral leaflets very unequal; unifoliolate leaves ovate or broadly ovate, 8–13.7 × 5–11 cm, apex acuminate, base cordate with lobes equal, rounded and rarely acute; leaves leathery, margin spinous-serrate, adaxially deep green, glossy, abaxially glaucous, papillose, and pubescent along veins, especially dense at the insertion of petioles and petiolules. Flowering stem with 1 trifoliolate leaf or 2 opposite leaves. Inflorescence simple, racemose, 3–14-flowered, 12–25 cm long, glandular. Pedicel 1–2.5 cm long, glandular. Flowers large, ca. 4 cm in diam. Outer sepals green or purplish, outer pair ovate-oblong, 3–5 × 2 mm, apex obtuse, inner pair broadly ovate, 4–5.5 × 3–4.5 mm, apex acuminate; inner sepals white, white tinged with rose, or rose, narrowly elliptic, 11–20 × 4–7 mm, apex acute. Petals subequal to or longer than the inner sepals, white or pale yellow with base yellow or orange, white with base rose or deep rose, rose, deep rose or pale purple, horn-shaped, 15–26 mm, tapering from a swollen but lamina-less base. Stamens ca. 4 mm long; anthers, yellow or green, ca. 3 mm long. Follicles oblong, 8–18 mm long; style rostriform. Seeds numerous.

#### Distribution and habitat.

*Epimediumleptorrhizum* is distributed in the montane forests or thickets in Guizhou, Hubei, Hunan and Chongqing, in elevations ranging from 350 m to 2100 m (Fig. 4).

#### Phenology.

*Epimediumleptorrhizum* flowers from April to May, and fruits from May to June.

#### Taxonomical remarks.

**Figure 3.**
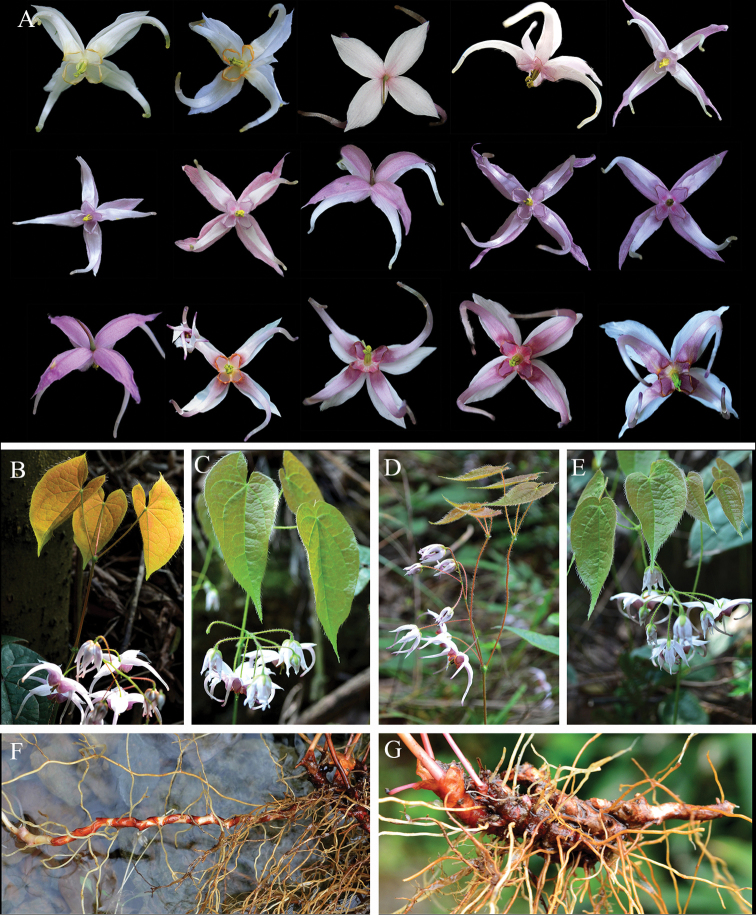
The variations and morphological characters of *E.leptorrhizum*. **A** the colour variations of inner sepals and petals **B–E** variations of the number and arrangement of stem-leaves **F** long creeping rhizome **G** stout and compact rhizome.

Abundant and continuous flower colour variation has been observed and illustrated for this species. The inner sepals presented continuous variation from white, white tinged with rose, through to completely rose. Petals varied from white or pale yellow with base yellow or orange, white with base rose or deep rose to completely rose, deep rose or pale purple (Fig. 3). Among them, white and pale yellow were only observed in one individual of the GZGY population. However, it is very common that the colour ranged from pale rose or rose to deep rose or pale purple among and within populations. The synonym, *E.brachyrrhizum* (GZLC) didn’t show any variation of flower colour, with rose inner sepals and pale rose petals.

In addition, we showed that the rhizome of *E.leptorrhizum* is usually long-creeping, occasionally compact. The flowering stem is usually with 1 trifoliolate leaf or 2 opposite leaves.

#### Additional specimens examined.

CHINA. **Guizhou**: Mt. Qianling, Guiyang, 10 March 1957, *Anon. 0123-635* (IMD); Mt. Qianling, Guiyang, 28 August 1977, *Anon. 5507* (IMD); Qianling Park, Guiyang, 1400 m, 19 March 1959, *Qiannan Exped. 0077* (PE); Guiyang, 5 June 1994, *B. L. Guo 94018* (IMD); Shuikousi, Guiyang, 1145 m, 2 April 2012, *Y. Q. Xu & Y. Xu 2012001* (JXCM); Minzhu, Longli, 1407 m, 1 April 2004, *B. L. Guo A38* (IMD); Longli, 1268 m, 15 April 2003, *B. L. Guo A81* (IMD); Ganlong, Songtao, 775 m, 19 April 2003, *B. L. Guo A87* (IMD); Songtao,19 April 2003, *B. L. Guo A98* (IMD); Huguo Temple, Yinjiang, 1100 m, 8 April 1964, *Z. S. Zhang & T. L. Min 401219* (PE, HGAS); Pingba, Anshun, 1500 m, 24 September 1959, *Anshun Exped. 1548* (PE); Ganwangou, Jiangkou, 350 m, 16 April 1990, *S. Z. He 90005* (HGAS); Wenang, Libo, 850 m, 29 April 1984, *Q. H. Chen 2377* (HGAS); Fuli, Bijie, 2100 m, 12 July 1959, *Bijie Exped. 266* (HGAS); Zhennan, Wuchuan, 580 m, 26 May 1977, *J. H. Yuan 001* (HGAS); Zhuoshui, Wuchuan, 580 m, 27 May 1977, *J. H. Yuan 002* (HGAS); Mt. Fanjing (Fanjingshan), Yinjaing, 1060 m-1200 m, 18 June 1963, *Anon. 680* (HGAS); Ganlong, Songtao, 907 m, 10 April 2013, *Y. Q. Xu & K. N. Zhang 2012010* (JXCM); Muhuang, Yinjiang, 1300 m, 1 April 2017, *Y. M. He & L. J. Liu 2017002* (JXCM). **Hubei**: Maoping, Jianshi, Enshi, 1019 m, 9 April 2008, *B. L. Guo & J. J. Liu 0804* (IMD); Maotian, Jianshi, Enshi, 983 m, 9 April 2008, *B. L. Guo & J. J. Liu 0805* (IMD); Banqiao, Enshi, 1258 m, 12 April 2008, *B. L. Guo & J. J. Liu 0808* (IMD); Daijing, Lichuan, Enshi, 1100 m, 25 April 1989, *B. L. Guo & X. Z. Luo 89005* (IMD); Mt. Fubao (Fubaoshan), Lichuan, Enshi, 1460 m, 29 April 1989, *B. L. Guo & X. Z. Luo 89009* (IMD); Mt. Fubao (Fubaoshan), Lichuan, Enshi, 1293 m, 8 April 2004, *B. L. Guo A54* (IMD); Lichuan, Enshi, 1141 m, 8 April 2004, *B. L. Guo A47* and *B. L. Guo A52* (IMD); Enshi, November 1958, *H. J. Li 8738* (PE); Qianping, Xintang, Enshi, 1523 m, 24 April 2012, *Y. Q. Xu 2012004* (JXCM); Mt. Fubao (Fubaoshan), Lichuan, Enshi, 1386 m, 2 April 2012, *Y. Q. Xu & Y. Xu 2012006* (JXCM). **Hunan**: Jishou University, Jishou (cultivated, from Longshan), 17 April 2003, *B. L. Guo A99* (IMD); Mt. Tianping (Tianpingshan), Sangzhi, 441 m, 5 April 2004, *B. L. Guo A32* (IMD); Mt. Tianping (Tianpingshan), Sangzhi, 26 August 1988, *H. N. Xun 3967* (PE). **Chongqing**: Xinglong, Youyang, 1000 m, 4 May 1959, *Anon. 02265* (PE); Guanyang, Wushan, 1056-1124 m, 9 May 2008, *Sanxia Exped. 1305* (PE). **Guangxi**: Dongshan, Quanxian, 840 m, 17 July 1958, *Y. C. Chen 00102* (IBK). **Zhejiang**: Datianping, Longquan, 1175 m, 9 April 2005, *B. L. Guo 0516* (IMD); Datianping, Longquan, 1210 m, 27 April 2018, *Y. Q. Xu & H. Huang 2018005* (JXCM).

**Figure 4.**
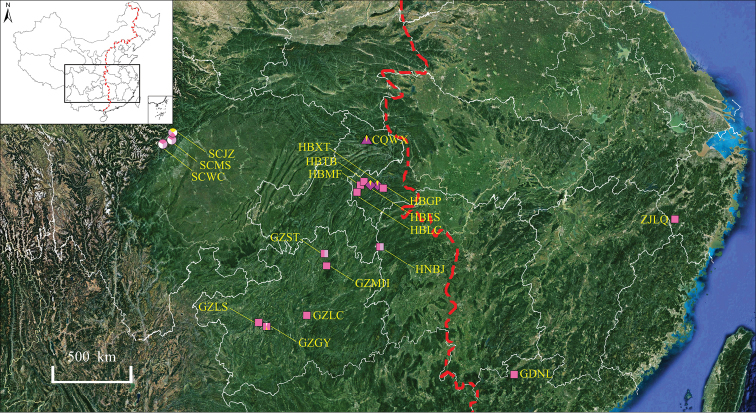
Geographical variation in flower colour patterns within *E.leptorrhizum* (boxes), *E.pauciflorum* (circles), *E.mikinorii* (diamonds) and *E.glandulosopilosum* (triangle). The colour of the boxes, circles, diamonds and triangle represent flower colour. The population codes are shown in Table 1. The left area of the red dotted line is western China.

### 3. Epimedium pauciflorum

Taxon classificationPlantaeRanunculalesBerberidaceae

K.C.Yen, Guihaia 14: 124. 1994

Fig. 5

Epimedium platypetalum var. tenuis B.L.Guo & P.G.Hsiao, Acta Phytotaxon. Sin. 31: 195. 1993. Type: CHINA. Sichuan: Shuixicun, Mao Xian, 1990 m, *H. R. Xie 89023*, (holotype: IMD! without accession number or barcode).

#### Type.

CHINA. Sichuan: Dagou, Maowen, 1700 m, *K. C. Yen & S. L. Shao 66535* (holotype: GXMI, barcode GXMI004519!; isotype: PE, barcode PE01432167!).

#### Description.

Herbs 6–30 cm tall. Rhizome long creeping, 1–3 mm in diam.; sometimes creeping to 20–25 cm. Leaves basal and cauline, trifoliolate; leaflets ovate or suborbicular, 1.3–4.5 × 1.2–3.5 cm, abaxially sparsely or occasionally densely puberulent, adaxially glabrous when mature, base deeply cordate with rounded lobes nearly touching, those of lateral leaflets conspicuously unequal, margin coarsely serrate, apex acute or shortly acuminate. Flowering stem with 1 trifoliolate, occasionally 1 unifoliolate leaf or 2 alternate trifoliolate leaves. Inflorescence simple, racemose, few-flowered (ca. 2–6 flowers), 3–13.5 cm long; inflorescence axis and pedicels glandular or pubescent. Pedicel 1–2.3 cm long; bracts ovate, 0.8–1.4 mm. Flowers ca. 2–2.5 cm in diam. Outer sepals fall sooner, greenish, caduceus, narrowly obovate, ca. 4 × 3 mm; inner sepals white, faintly rose-tinged or pale rose, broadly lanceolate, ca. 12 × 5 mm, apex obtuse. Petals declined, white, white tinged with pale rose, rose, pale yellow, pale rose with the base purple yellow, horn-shaped, longer than inner sepals, blunt spurs ca. 15 mm long, expanded at base into a lamina 6 mm high. Stamens ca. 4 mm long; anthers yellow, ca. 2.5 mm long. Follicles oblong, 10–15 cm long; style rostriform. Seeds 4–6.

#### Distribution and habitat.

*Epimediumpauciflorum* is only known from the mountains of Maowen (the holotype locality, once and now divided into Maoxian, Wenchuan and Lixian) in Sichuan province, usually occurring in forest edges and weedy slopes, at high elevations approximately 1700–2600 m (Fig. 4).

#### Phenology.

*Epimediumpauciflorum* flowers from April to May, and fruits also from April to May.

#### Taxonomical remarks.

**Figure 5.**
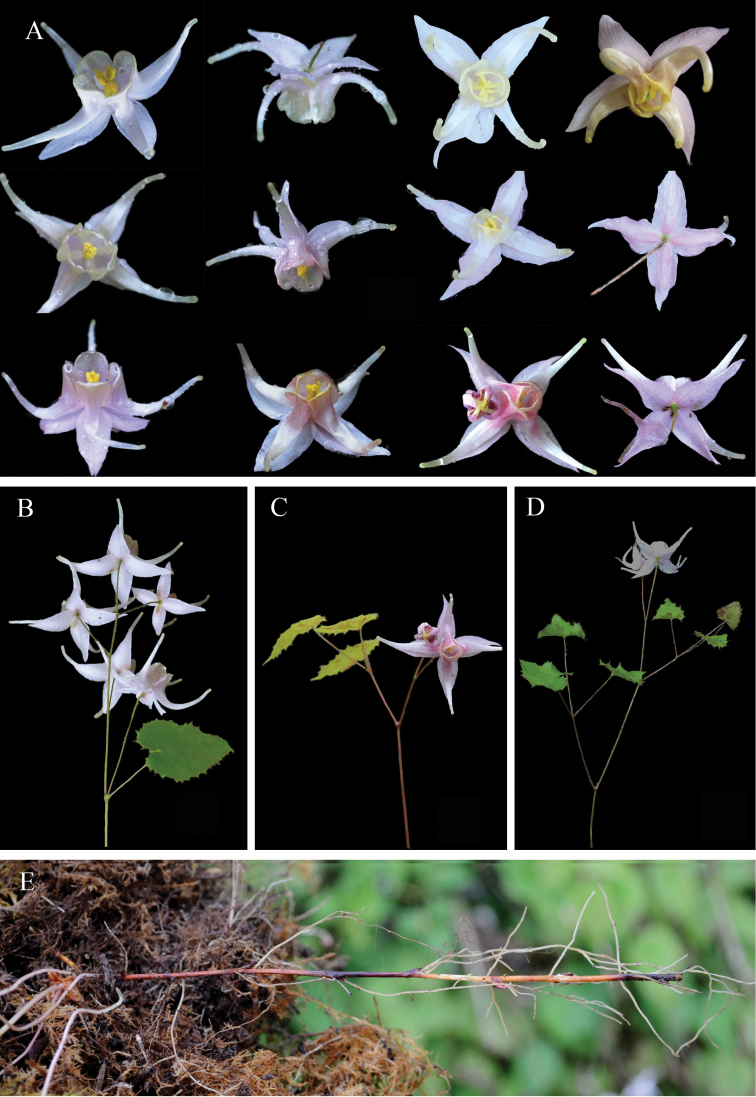
The variations and morphological characters of *E.pauciflorum*. **A** colour variations of inner sepals and petals **B–D** variations of the number and arrangement of stem-leaves **E** long creeping and thread-like rhizome.

*Epimediumpauciflorum* is a low-growing species with a few-flowered inflorescence. Some information about the colour variation was recorded in part of the specimens. The colours of three specimens (*Z. L. Kun 89022*, *B. L. Guo & W. K. Bao 97040*, *B. L. Guo 88182*) were described as white, while the other two (*B. L. Guo & W. K. Bao 97031* and *97032*) were described as yellow. We observed a variety of colour variations; the inner sepals were white, faintly purple-tinged or pale rose, whereas the petals were white, white tinged with pale rose, rose, pale yellow, pale rose with the base purple yellow (Fig. 5). Among them, the pale-yellow flowers were only observed in SCJZ population. The specimens (*B. L. Guo & W. K. Bao 97031* and *97032*) that were with yellow flowers and the SCJZ population in this study were sampled from the same location.

All the examined specimens were with 1 trifoliolate leaf on the flowering stem uniformly. But 1 unifoliolate leaf (only in SCWC population) or 2 alternate trifoliolate leaves (only in SCJZ population) (Fig. 5) were also observed in our field investigation.

#### Additional specimens examined.

CHINA. **Sichuan**: Jingzhou, Maoxian, 1700 m, 16 April 1987, *K. C. Yen & S. L. Shao 66535* (PE, GXMI); Jingzhou, Maoxian, 1770 m, 19 April 1997, *B. L. Guo & W. K. Bao 97031* and *B. L. Guo & W. K. Bao 97032* (IMD); Jingzhou, Maoxian, 1770 m, 19 April 1997, *B. L. Guo & W. K. Bao 97040* (IMD); Jingzhou, Maoxian, 1800 m, 2 May 1989, *Z. L. Kun 89022* (IMD); Shuishi, Maoxian, 2000 m, 19 May 1989, *H. R. Xie 89023* (IMD); Jingzhou, Maoxian, 1800 m, 3 September 1988, *B. L. Guo 88182* (IMD); Daheba, Maoxian, 1800 m, 6 September 1988, *B. L. Guo 88195* (IMD); Mt. Bianshi (Bianshishan), Maowen, 2600 m, 22 August 1985, *F. Li 822-1* (IMD); Mt. Bianshi (Bianshishan), Maowen, 2600 m, 23 August 1985, *F. Li 823-1* (IMD); Weizhou, Wenchuan, 2 May 1959, *Maowen Exped. 2046* (PE, CDBI); Miansi, Wenchuan, 1780 m, 12 April 2017, *Y. M. He et al. 2017007* (JXCM); Fengyi, Maoxian, 1800 m, 13 April 2017, *Y. M. He et al. 2017009* (JXCM). ENGLAND: cultivated at Blackthorn Nussery (from Maoxian, *Ogisu 92020*), *W. T. Stearn* (PE).

### 4. Epimedium mikinorii

Taxon classificationPlantaeRanunculalesBerberidaceae

Stearn, Kew Bull. 53: 214. 1998

Fig. 6

#### Type.

CHINA. Hubei: Enshi, 670 m, April 1995, *Ogisu 95039*, cultivated at Blackthorn Nursery, Kilmeston, Hampshire; collected by W.T.Stearn, 5 April 1997 (holotype: K, not seem).

#### Description.

Herbs 26–94 cm tall. Rhizome compact. Leaves basal and cauline, trifoliolate; leaflets adaxially dark green, lanceolate, 8–17.4 × 3–8.6 cm, leathery, abaxially glaucous, glabrous or with appressed hairs, base cordate with equal lobes rounded, those of lateral leaflets oblique with outer lobe large and acute, inner lobe smaller and rounded, margin closely spinose-serrulate, apex long acuminate. Flowering stem with 2 opposite or occasionally 3-whorled trifoliolate leaves. Inflorescence compound, loose, 8–50(–85)-flowered, ca. 7–30 cm long, glabrous, with lower peduncles 3–5-flowered. Pedicel 1.0–1.5 cm long, glabrous. Flowers ca. 2.5–3.5 cm in diam. Outer sepals oblong, 4–6 × 2–4 mm; inner sepals yellowish, white, rose-tinged or rose, elliptic, 11–16 × 7–12 mm. Petals longer and much narrower than inner sepals, yellow, orange, purple or purple with yellow-edged lamina ca. 3.5 mm high; spur slightly curved or almost straight, subulate, elongated, 15–20 mm long. Stamens ca. 4 mm long; anthers yellow or green, ca. 3 mm long. Follicles oblong, 8–15 mm long; style rostriform. Seeds numerous.

#### Distribution and habitat.

*Epimediummikinorii* is restricted to the mountains of Hubei (Enshi), Western of China, usually occurring at elevations ranging from 500 m to 1700 m (Fig. 4).

#### Phenology.

*Epimediummikinorii* flowers from April to June, and fruits from May to June.

#### Taxonomical remarks.

The field investigation found extensive colour variations. The inner sepals have different colours, ranging from white, rose-tinged, purple-tinged, through to rose and pale purple. Petals also exhibited abundant colour variation, such as yellow, orange, purple, purple but laminae yellow-edged or yellow at both ends while purple in the middle. Although the specimens of *E.mikinorii* that can be referred to are very limited, the characters and the descriptions of some herbarium specimens were consistent with our observations. Some specimens from Enshi had yellow flowers (*B. L. Guo & X. Z. Luo 89012*) or the flower colour varied from pale yellow to pale purple and purple (*B. L. Guo A29, B. L. Guo A50*) or from yellow to orange and pale purple (*B. L. Guo & J. J. Liu 0810*).

**Figure 6.**
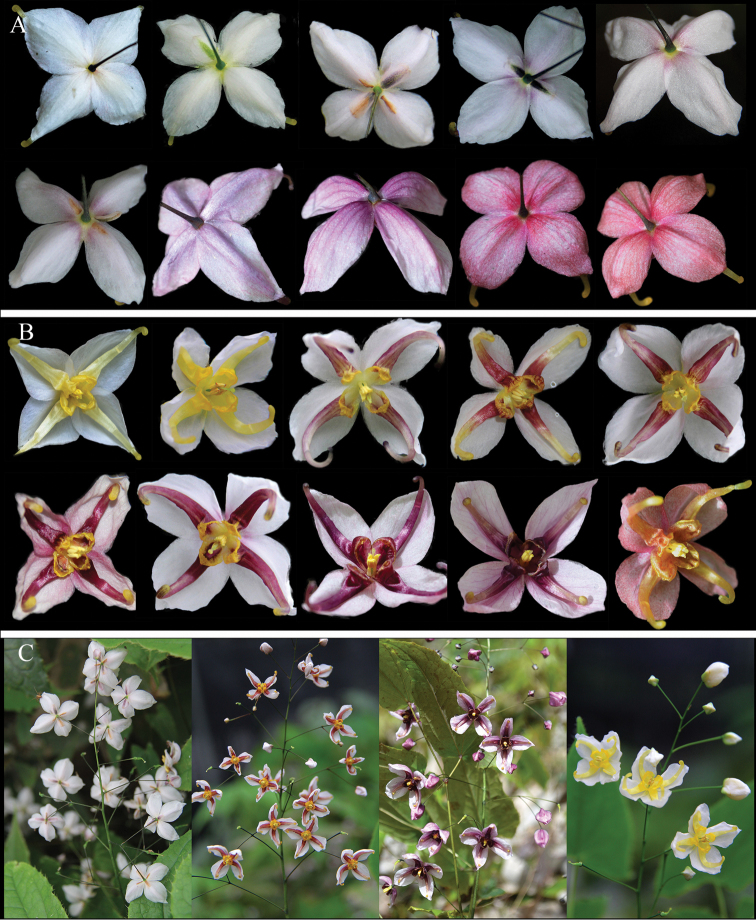
The variations and morphological characters of *E.mikinorii*. **A** colour variations of inner sepals **B** colour variations of petals **C** individuals that with different flower colour.

According to our field investigation and the common garden experiment on two populations, parts of the spurs were almost straight, while most were slightly curved (Fig. 6). The petals, in fact, presented a continuous variation from much longer to slightly longer than the inner sepals (Fig. 6).

As far as the indumentum of leaves concerned, we observed that approximately 40% of the individuals from HBES (near its type locality) and some individuals from HBXT were with white appressed hairs abaxially. Moreover, all the previous descriptions and specimens on the flowering stem of *E.mikinorii* were with 2 opposite trifoliolate leaves. However, 3-whorled trifoliolate was also observed in the HBES population (*S. X. Liu et al. 2016017*).

#### Additional specimens examined.

CHINA. **Hubei**: Baiguo, Enshi, 754 m, 21 April 2016, *S. X. Liu et al. 2016017* (JXCM); Xiashuba, Baiguo, Enshi, 500 m, 4 May 1989, *B. L. Guo & X. Z. Luo 89012* (IMD); Baiguo, Enshi, 852 m, 8 April 2004, *B. L. Guo A29* (IMD); Jinlong, Dengta, Enshi, 1100 m, 22 April 1974, *X. S. Zou 74001* (HIB); Dongliushui, Dengta, Enshi, 900 m, 25 April 1974, *X. S. Zou 74006* (HIB); Xintang, Enshi, 1100 m, 5 December 2004, *Y. J. Zhang & X. D. Li 011* (HIB); Baiguoping, Enshi, 970 m, 5 December 2004, *Y. J. Zhang & X. D. Li 017* (HIB); Xintang, Enshi, 985 m, 6 December 2004, *Y. J. Zhang & X. D. Li 019* (HIB); Xintang, Enshi, 2 June 1974, *Y. J. Ma 277* (HIB); Huangjindong, Xianfeng, 829 m, 7 April 2004, *B. L. Guo A48* (IMD); Longfeng, Enshi, 533 m, 8 April 2004, *B. L. Guo A50* (IMD); Changlinggang, Shaunghe, Enshi, 1700 m, 20 August 2005, *B. L. Guo 05118* (IMD); Luzhuba, Baiyangping, Enshi, 635 m, 9 April 2008, *B. L. Guo & J. J. Liu 0807* (IMD); Shaziba, Sancha, Enshi, 944 m, 12 April 2008, *B. L. Guo & J. J. Liu 0809* (IMD); Changlinggang, Enshi, 1677 m, 13 April 2008, *B. L. Guo & J. J. Liu 0810* (IMD).

### 5. Epimedium glandulosopilosum

Taxon classificationPlantaeRanunculalesBerberidaceae

H.R.Liang, Acta Phytotaxon. Sin. 28: 323. 1990

Fig. 7

#### Type.

CHINA. Chongqing: Wushan, 850 m, 25 April 1987, *H. R. Liang 144* (holotype: BCMM, lost); Chongqing: Wushan, 1000 m, 19 April 1989, *B. L. Guo 89003* (neotype, designated by Zhang et al. 2011: IMD! without accession number or barcode).

#### Description.

Herbs 16–80 cm tall. Rhizome long creeping or compact, 1–5 mm in diam., internodes can be more than 10 cm. Leaves basal and cauline, usually trifoliolate or occasionally unifoliolate; leaflets of trifoliolate leaves narrowly ovate or lanceolate, 4.6–15.3 × 2.4–7.6 cm, apex acuminate, base deeply or shallowly cordate with a narrow sinus, terminal leaflet with equal and obtuse lobes, lateral leaflets conspicuously oblique with inner lobe small and obtuse, outer lobe larger and obtuse, acute or acuminate. Unifoliolate leaves ovate, broadly ovate or lanceolate, 5.0–13.0 × 2.5–6.5 cm, apex acuminate, base deeply cordate with lobes equal and obtuse or acute. Leaves coriaceous, margin spinous-serrate, adaxially deep green, obtuse, abaxially covered with villi. Petiolule, petiole and flowering stem with multicellular glandular hairs and villi, which are densest at nodes. Flowering stems usually have 2 opposite trifoliolate leaves, sometimes with 3 whorled trifoliolate leaves, 1 unifoliolate or trifoliolate leaf, rarely with 2 opposite unifoliolate or 2 leaves (alternate or opposite) with one trifoliolate and the other unifoliolate. Inflorescence racemose or compound with 8–24(–36)-flowered, 9.6–16 cm long; inflorescence axis and pedicels glandular pubescent. Pedicel 1–3 cm long. Flowers, ca. 3 cm, pale yellow, pale purple or purple. Sepals 8 in 2 whorls; outer sepals ovate, ca. 3.5 × 2 mm, red-purple; inner sepals narrowly ovate, 8–10 × 4–6 mm, white to faintly pink. Petals spurred without lamina, pale yellow, pale purple or purple, horn-shaped, 13–20 mm long. Stamens ca. 4 mm long; anthers yellow or green, ca. 3 mm long. Follicles oblong, 12–19 mm long; style rostriform. Seeds numerous.

#### Distribution and habitat.

Endemic to Chongqing, Western of China, usually occurs in forests or thickets. The elevations ranged from 850 m to 1160 m (Fig. 4).

#### Phenology.

*Epimediumglandulosopilosum* flowers from April to May, and fruits from May to June.

#### Taxonomical remarks.

**Figure 7.**
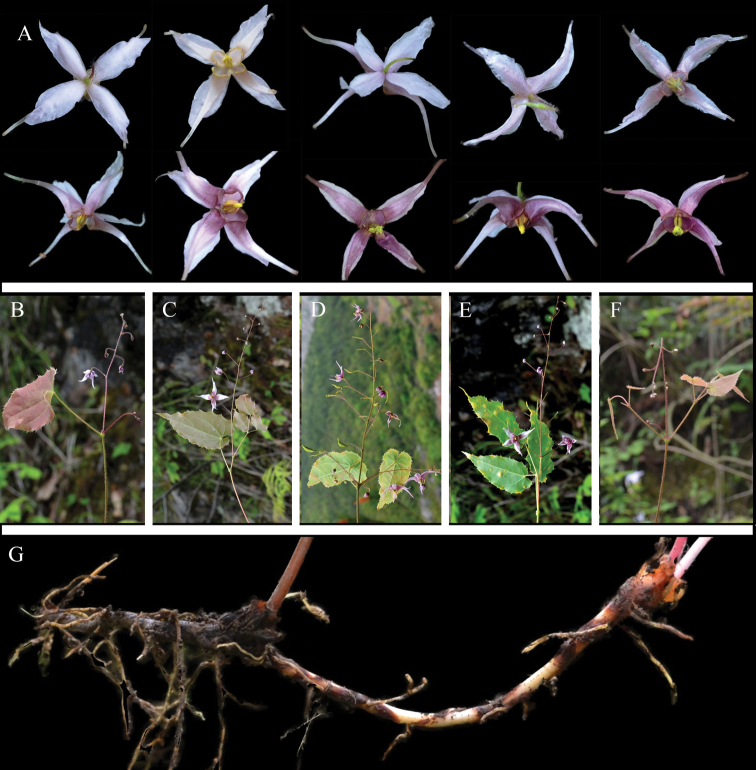
The variations and morphological characters of *E.glandulosopilosum*. **A** variations of flower colour **B–F** variations of the number and arrangement of stem-leaves **G** creeping and slender rhizome.

The observations of the present study showed that only several individuals had yellowish flowers, while the rest had pale purple or purple flowers (Fig. 7). Only two specimens of *E.glandulosopilosum* are available for reference, but the specimens showed similar results. *B. L. Guo & X. Z. Luo 89003* described the flower colour as whitish to pale purple, rarely yellow, while *B. L. Guo A15* described it as pale purple or pale yellow.

The protologue and the *Flora of China* described *E.glandulosopilosum* as “outer sepals narrowly ovate, 8–9 × 4–5 mm, inner sepals similar to outer sepals in size and shape” (Liang et al. 1990; Ying et al. 2011). However, according to our observation in the field and cultivation, the outer sepals (ovate, 3.5 × 2 mm) were obviously narrower and shorter than inner sepals (narrowly ovate, 8–10 × 4–6 mm).

In the present study, all individuals from the population CQWX (Wuxi) were with long creeping and slender rhizomes (1–3 mm in diam.) (Fig. 7; *S. X. Liu et al. 2016018*). The herbarium specimens from Wushan (*B. L. Guo & X. Z. Luo 89003*) and Wuxi (*B. L. Guo A15*) also presented creeping rhizome. The rhizome was therefore revised as long creeping or compact.

The leaves of some individuals were densely covered with golden-yellow villi on the abaxial side. And the petiolule, petiole and flowering stem were covered with multicellular glandular-hairs and golden-yellow villous, which are densest at the nodes. However, these indumentum characters were not stable and exhibited great variation in colour and thickness. Depending on the individuals and/or developmental stage (young or old), the indumentum of the abaxial villi varied continuously from dense to sparse, and the colour was also not always typical golden-yellow, ranging from white to yellow.

Our field investigation showed that the flowering stem primarily had 2 opposite trifoliolate leaves. In addition, abundant variations, such as 1 unifoliolate or trifoliolate leaf, 2 opposite unifoliolate leaves or 2 alternate leaves with one trifoliolate and the other unifoliolate, were also observed. And all these styles were presented in two specimens (Wushan: *B. L. Guo & X. Z. Luo 89003* and Wuxi: *B. L. Guo A15*).

#### Additional specimens examined.

CHINA. **Chongqing**: Tongcheng, Wuxi, 1159 m, 14 April 2004, *B. L. Guo A15* (IMD); Tongcheng, Wuxi, 1161 m, 109°46', 31°23', 23 April 2016, *S. X. Liu et al. 2016018* (JXCM); Guandu, Wushan, 1000 m, 19 April 1989, *B. L. Guo & X. Z. Luo 89003* (IMD).

Key to the species of *Epimedium* in China

In total, 57 species and 6 varieties have been described from China, although 16 of these were designated as synonyms. Epimediumplatypetalumvar.tenue B.L.Guo & P.K.Hsiao was treated as synonym of *E.pauciflorum* (Stearn 2002). *Epimediumsimplicifolium* T.S.Ying and *E.chlorandrum* Stearn were treated as synonyms of *E.acuminatum* (Zhang et al. 2011; Zhang et al. 2015). Epimediumsagittatumvar.guizhouense S.Z.He & B.L.Guo and *E.pudingense* S.Z.He, Y.Y.Wang & B.L.Guo were treated as a synonym of *E.sagittatum* (Sieb. &Zucc.) Maxim. and E.sagittatumvar.glabratum T.S.Ying, respectively (Xu et al. 2014b). *Epimediumcoactum* H.R.Liang et W.M.Yan, E.coactumvar.longtouhum H.R.Liang, E.myrianthumvar.jianheense S.Z.He et B.L.Guo, *E.multiflorum* Ying and *E.jingzhouense* G.H.Xia & G.Y.Li were integrated into *E.myrianthum* Stearn (Xu et al. 2014b). *Epimediumrhizomatosum* Stearn, *E.brachyrrhizum* Stearn, *E.dewuense* S.Z.He, Probst et W.F.Xu, E.sagittatumvar.oblongifoliolatum were treated as synonyms of *E.membranaceum* Stearn, *E.leptorrhizum*, *E.dolihostemon* Stearn and *E.borealiguizhouense* S.Z.He & Y.K.Yang, respectively (Zhang et al. 2015). *Epimediumlishihchenii* Stearn and *E.baojingense* Q.L.Chen & B.M.Yang were treated as subspecies and variety of *E.franchetii* Stearn, respectively (Liu et al. 2016). Additionally, *Epimediumtianmenshanense* T.Deng, D.G.Zhang & H.Sun is an insufficiently known taxon, due to the petals being found to be highly variable in morphology (both shape and size) (our field observation). Therefore, 45 species, 1 subspecies (E.franchetiissp.lishihchenii) and 2 varieties (E.sagittatumvar.glabratum T.S.Ying, E.franchetiivar.baojingense) are recognised.

### Key to the species of *Epimedium* in China

**Table:** 

1	Leaves always unifoliolate	**2**
–	Leaves usually trifoliolate, leaflets 3 or 9, sometimes 7 or 5, occasionally unifoliolate	**3**
2	Rhizome long creeping, 1.5–3 mm diam.; flowering stem usually with 1 leaf, occasionally with 2 leaves; leaves cordate, 2.5–4 × 3–4 cm, abaxially sparsely pubescent; inflorescence raceme or panicle; inner sepal light pink, 4.6–6 × 1.8–3.2 mm; spurs orange red, saccate, 1.5–2 mm long	***E.elachyphyllum* Stearn**
–	Rhizome compact, 2–5 mm diam.; flowering stem usually with 2 opposite leaves; leaves broadly ovate, 5–7 × 3–5 cm, glabrous on both surfaces; inflorescence panicle; inner sepal whitish, 4.5 ×2 mm; spurs yellowish brown, 2 mm long	***E.muhuangense* S.Z.He & Y.Y.Wang**
3	Petals flat or flat with slightly cucullate base; flowers campanulate, spurless, yellow	**4**
–	Petals saccate, slipper-shaped, or with spurs; flowers not campanulate, white, pink, purple or yellow	**7**
4	30–50 cm tall; rhizome compact; leaflets coriaceous; inflorescence panicle, 30–80-flowered, 30–35 cm long, glandular; flower ca. 7 mm in diam.; petals flat with slightly cucullate base	***E.reticulatum* C.Y.Wu ex S.Y.Bao**
–	20–50 cm tall; rhizome compact or long creeping; leaflets membranous; inflorescence raceme or panicle, 2–43-flowered, 7–23 cm long, glabrous or glandular; flower ca. 10 mm in diam.; petals flat	**5**
5	35–50 cm tall; rhizome always compact, 4–6 mm in diam.; leaflets ovate, 4.5–6 × 2.5–4 cm; inflorescence panicle, 15–43-flowered, 11–23 cm long, glabrous; inner sepals red-tinged; petals spurless, obovate, 6–8 × 5–7 mm	***E.campanulatum* Ogisu**
–	20–40 cm tall; rhizome long creeping or compact, 1–3 mm in diam.; leaflets subrounded or ovate, 2.5–4.5 × 2–4 cm; inflorescence raceme, 2–14-flowered, 7–16 cm long, glandular; inner sepals purple-red; petals spurless, oblong or obovate, 6–8 × 4–5 mm	**6**
6	20–40 cm tall; rhizome always long creeping; leaves trifoliolate, 5-foliolate, sometimes 7-foliolate, leaflets ovate, 2.5–4 × 2–3 cm; inflorescence usually 7–14-flowered, 10–16 cm long, glandular; pedicels 1–2 cm long, glandular; out sepals pale purple, broadly ovate, 4 × 1.5 mm; inner sepals elliptic, 5 × 1.5 mm	***E.ecalcaratum* G.Y.Zhong**
–	25–35 cm tall; rhizome compact or long creeping; leaves always trifoliolate, leaflets subrounded, 4.5 × 4 cm; inflorescence usually 2–6-flowered, 7–12 cm long, pedicels 0.5–1 cm long, out sepals green, triangular-lanceolate, 2 × 1 mm; inner sepals ovate, 4 × 1.5 mm	***E.platypetalum* K.Meyer**
7	Petals with elongated curved spurs and basal laminae	**8**
–	Petals with long slender spurs but without lamina, or petals very short	**23**
8	Leaves on flower stem biternate (leaflets 9), or 7-, 5-, 3-foliolate	**9**
–	Leaves on flower stem always 3-foliolate, occasionally unifoliolate	**12**
9	15–40 cm tall; rhizome long creeping; flowering stem always with 1 biternate leaf, leaflets ovate, 3–13 × 2–8 cm; inflorescence raceme, usually 3–8-flowered, 6–12 cm long, glabrous; pedicels 1–2 cm long, glabrous; flower yellowish or white, 2.5–3 cm in diam.; inner sepals narrowly ovate to lanceolate, 8–10 × 3–4 mm; spurs 10–15 mm; laminae ca. 6 mm high	***E.koreanum* Nakai**
–	25–60 cm tall; rhizome compact; flowering stem usually with 2–4 leaves or 1 leaf (5, 3-foliolate); inflorescence raceme or panicle with 2–5-flowered peduncles below, usually 4–60-flowered, 6–32 cm long, glandular; pedicels 1.5–3 cm long, glandular; flower pale yellow or pale sulphur yellow, 2–4 cm in diam.; spurs 10–19 mm; laminae 6–13 mm high	**10**
10	25–44 cm tall; flowering stem usually with 1 leaf or 2–3 leaves (5-, 3-foliolate); inflorescence raceme; usually 4–18-flowered, 6–17 cm long; inner sepals yellow, 11 × 4 mm; spur 10–14 mm	***E.flavum* Stearn**
–	33–60 cm tall; flowering stem with 2–4 leaves (biternate or 7-, 5-, 3-foliolate); inflorescence panicle with 2–5-flowered peduncles below; usually 11–60-flowered, 14–32 cm long; inner sepals purplish red, 4–6.5 × 2–4 mm; spur 10–19 mm	**11**
11	50–60 cm tall; flowering stem usually with 3 leaves, biternate, or leaflets 7, 5, 3; inflorescence 20–60-flowered, 20–30 cm long; petal spurs horizontally spreading, spurs 17–19 mm; laminae 6–7 mm high	***E.xichangense* Y.J.Zhang**
–	33–60 cm tall; flowering stem usually with 2 leaves, leaflets 5 or 3; inflorescence 11–44-flowered, 14–32 cm long; petal spurs downward-curved, spurs 10–15 mm; laminae 7–13 mm high	***E.davidii* Franch.**
12	Rhizome long creeping or compact; inflorescences usually raceme, occasionally compound below	**13**
–	Rhizome always compact; inflorescences always panicle	**20**
13	Petals slightly shorter or nearly as long as inner sepals	**14**
–	Petals longer than inner sepals	**15**
14	25–35 cm tall; rhizome long creeping, 1 mm in diam.; leaflet ovate or narrowly ovate, 3–6 × 1–3 cm, almost glabrous or with scattered hairs; inflorescence 3–12-flowered, 12–14 cm long; pedicels 2–3 cm long, glabrous; flower 2.5 cm in diam., inner sepals white, spurs white, 15–18 mm, nearly as long as inner sepals; laminae 7–8 mm high	***E.ogisui* Stearn**
–	18 cm (or longer) tall; rhizome compact, 4.5–8 mm in diam.; leaflet narrowly ovate, 6.5–11.2 × 3.7–6.1 cm, with scattered hairs; inflorescence 8–22-flowered, 10–17 cm long; pedicels 2–2.7 cm long, glabrous or sometime glandular; flower 3–3.8 cm in diam., inner sepals white, spurs deep purple, 15–17 mm, shorter or nearly as long as inner sepals; laminae 5–6 mm high	***E.shennongjiaensis* Y.J.Zhang & J.Q.Li**
15	Flowers large, ca. 4–5 cm in diam	**16**
–	Flowers less than 4 cm in diam	**17**
16	30 cm tall; leaflet narrowly ovate, 6–9 × 2.5–4 cm, with scattered, short, erect hairs; inflorescence raceme, 8-flowered, ca. 20 cm long; pedicels 2.5–5 cm long, glabrous; flower 4–5 cm in diam., inner sepals white, elliptic, 16 × 8–9 mm; spurs white, slight yellowish or purple-tinged at base, 25 mm, elongated; laminae 7 mm high	***E.latisepalum* Stearn**
–	25 cm tall; leaflet narrowly ovate, 4–8 × 2.5–5.5 cm, almost glabrous, or with scattered, appressed hairs; inflorescence raceme, 6–10-flowered, ca. 13 cm long; pedicels 2 cm long, glabrous; flower 4.5 cm in diam., inner sepals reddish, cymbiform, 6 × 2.5 mm; spurs pale yellow, 22 mm, elongated; laminae 10 mm high	***E.fangii* Stearn**
17	Flowering stem usually with 1 trifoliolate, occasionally 1 unifoliolate leaf or 2 alternate trifoliolate leaves; 3–6-flowered	***E.pauciflorum* K.C.Yen**.
–	Flowering stem with 2 opposite trifoliolate leaves, occasionally 1 trifoliolate leaves or 3 whorled leaves; 6–18-flowered	**18**
18	Rhizome always long creeping, 1.5–2.5 mm in diam.; leaflet narrowly ovate, 4–5.5 × 2–2.5 cm, abaxially with appressed hairs; inflorescence always raceme, 6–9-flowered, 6–7 cm long; pedicels 1–1.5 cm long, glabrous; flower 1.5–2 cm in diam., inner sepals red, ovate-oblong, 5 × 2–3 mm; petals yellow, spur subulate, obviously curved, 12 mm long, much longer inner sepals; laminae 7 mm high	***E.shuichengense* S.Z.He**
–	Rhizome compact or long creeping, 2.1–5.6 mm in diam.; leaflet narrowly ovate, 6.8–19 × 2.5–6.3 cm, almost glabrous; inflorescence raceme or sometimes compound with 3-flowered peduncles below, 6–18-flowered, 8–15 cm long; pedicels 1.5–3 cm long, glabrous or glandular; flower 3–3.5 cm in diam., inner sepals white to pinkish, ovate, 11–14 × 5–10 mm; spur slightly curved, 15–16 mm long, a little longer inner sepals; laminae 5–7 mm high	**19**
19	12–68 cm tall; rhizome long creeping or compact; leaflet narrowly ovate, 6.8–13.5 × 2.9–6.3 cm, almost glabrous or with minute hairs; flower stem with 2 opposite trifoliolate leaves or 1 trifoliolate leaf; flower ca. 3 cm in diam., inner sepals white to pale pink; petals purple, laminae 5 mm high	***E.epsteinii* Stearn**
–	15–25 cm tall; rhizome long always compact; leaflet narrowly ovate, 7–19 × 2.5–5.5 cm, glabrous; flower stem with 2 opposite trifoliolate leaves; flower 3–3.5 cm in diam., inner sepals pinkish-lilac; petals chestnut-brown, spur pale greenish, laminae 7 mm high	***E.stearnii* Ogisu & Rix**
20	Flower yellow or pale yellow, 3.5–4 cm in diam., petals with obvious basal laminae 7–8 mm high	**21**
–	Flower usually purple, occasionally yellow, 2.5–3 cm in diam., petals with slight basal laminae 2–3.5 mm high	**22**
21	Leaflet oblong-elliptic or narrowly ovate, 8.1–15.5 ×3.6–6.3 cm, almost glabrous or sparingly pubescent; inflorescence compound with 2–3-flowered peduncles below, 7–38-flowered, 9–24 cm long, almost glabrous; pedicels 1–2 cm, glandular; flower 3.5 cm in diam., inner sepals broadly elliptic, 5–6 × 3–4 mm, purplish red; petals yellow, spur 15–18 mm long, much longer inner sepals; laminae 8 mm high	***E.hunanense* (Hand.-Mazz.) Hand.-Mazz.**
–	Leaflet lanceolate or narrowly lanceolate, 6.4–23 × 1.8–6.3 cm, abaxially pubescent; inflorescence panicle, 9–70(–100)-flowered, 13–30 cm long, sparsely glandular or glabrous; pedicels 0.7–1.5 cm, almost glabrous; flower ca. 4 cm in diam., inner sepals ovate, 12 × 6–8 mm, milky white; petals pale yellow, spur 20 mm long, much longer inner sepals; laminae 7 mm high	***E.wushanense* T.S.Ying**
22	27–54 cm tall; leaflet narrowly ovate to narrowly lanceolate, 6–14.2 × 2–6.1 cm, abaxially sparingly pubescent; inflorescence panicle, 11–45-flowered, 13–22 cm long, glabrous; inner sepals ovate or broadly ovate, 8–13 × 4–8 mm; spurs of petals slightly longer than inner sepals, 10–15 mm long, laminae 2–3 mm high	***E.pseudowushanense* B.L.Guo**
–	26–94 cm tall; leaflet lanceolate, 8–17.4 × 3–8.6 cm, abaxially glabrous or sometimes sparingly pubescent; inflorescence panicle, 8–50(–85)-flowered, 7–30 cm long, glabrous; inner sepals elliptice, 11–16 × 7–12 mm; spurs of petals obviously longer than inner sepals, 15–20 mm long, laminae ca. 3.5 mm high	***E.mikinorii* Stearn**
23	Petals very short, 2–8 mm, and much shorter than the inner sepals	**24**
–	Petals with long slender spurs, 12–26 mm, without lamina	**33**
24	Rhizome always compact; stamens 7–10 mm long, conspicuous, protruding, filament much longer than anther	**25**
–	Rhizome long creeping or compact; stamens 2–6 mm long, filament equalling or much shorter than anther	**27**
25	Inner sepals always spreading, narrowly elliptic, 8–10 × 3–4.5 cm; petals cucullate, obviously incurved; filament yellowish, 4.5–5 mm long; anthers green, 2.5 mm long	***E.dolichostemon* Stearn**
–	Inner sepals reflexing or slightly reflexing, ovate or narrowly lanceolate, 11–18 × 2.5–5 cm; petals almost straight; filament purple or white, 4–5 mm long; anthers green or yellow, 2–4 mm long	**26**
26	Leaflets narrowly ovate, 6.5–13.5 × 2.7–5.7 cm, abaxially glabrous; inflorescences panicle, 19–69-flowered, 10–28 cm long, glabrous; inner sepals ovate, 11 × 5 mm; petals purple, straight, 4 mm long; filaments purple; anthers green	***E.qingchengshan* G.Y.Zhong &B.L.Guo**
–	Leaflets narrowly ovate, 7.5–14.4 × 2.3–6.4 cm, abaxially glabrous or sparsely pubescent; inflorescences panicle, 9–48-flowered, 9.2–28.6 cm long, glandular; inner sepals narrowly lanceolate, 15–18 × 2.5–3.5 mm; petals purple, with whitish tip, straight, 6.5–9.5 mm long; filaments purple or white; anthers green or yellow	***E.fargesii* Franch.**
27	Flower stem with 1 biternate leaf, 9 leaflets	***E.brevicornu* Maxim.**
–	Flower stem with 2 (occasionally 3) trifoliate leaves	**28**
28	Plant glabrous throughout; the two lateral leaflets not cordate at base but obliquely truncate	***E.truncatum* H.R.Liang**
–	Plant always with hairs; the leaflets all cordate at base	**29**
29	Inner sepals 5–12 mm long and flowers more than 10 mm in diam.; inflorescences glandular	**30**
–	Inner sepals 3–5 mm mm long and flowers 6–8(–10) mm in diam.; inflorescences usually glabrous	**31**
30	Rhizome always compact; leaflets ovate, 8–9 × 4–7 cm; inflorescences 20–40-flowered; inner sepals lanceolate, 12 × 3–3.5 mm; petals with slight laminae and blunt spur	***E.stellulatum* Stearn**
–	Rhizome compact, sometimes elongated; leaflets ovate, narrowly ovate or lanceolate, 3–15 × 2–8 cm; inflorescences many-flowered, usually more than 50 flowers; inner sepals narrowly lanceolate, 5–10 × 1.5–2.5 mm; petals with blunt spur, almost straight, saccate	***E.pubescens* Maxim.**
31	Inflorescences narrow and straight, 4–5 cm broad, 21–153-flowered; petals saccate, blunt, 2–4 mm long	***E.sagittatum* (Sieb. & Zucc.) Maxim.**
–	Inflorescences loose, 7–15 cm broad, 54–1140-flowered; petals slipper-shaped, 2–3 mm long	**32**
32	Leaflets narrowly ovate, 9.1–16.4 × 4.2–10.3 cm; inflorescences 7–9 cm broad, 54–572-flowered; outer sepals black or dark purple, petals orange-yellow and red	***E.myrianthum* Stearn**
–	Leaflets lanceolate or narrowly lanceolate, 13–18 × 2.5–4 cm; inflorescences usually 12–18 cm broad, 61–1142-flowered; outer sepals purplish, petals yellow	***E.borealiguizhouense* S.Z.He & Y.K.Yang**
33	Leaves on flower stem biternate (9 leaflets)	***E.elongatum* Komarov**
–	Leaves on flower stem with 3 leaflets, occasionally 1 leaflet	**34**
34	Inflorescences usually raceme	**35**
–	Inflorescences usually panicle	**40**
35	Leaflet 3.2–9.5 × 2.2–6 cm; flower 2–3 cm in diam.; inner sepals ovate, 5–7 × 2–4 mm	**36**
–	Leaflet 3–16 × 2–8.6 cm; flower 3–4.5 cm in diam.; inner sepals narrowly ovate, narrowly lanceolate or narrowly elliptic 8–20 × 3–7 mm	**37**
36	Rhizome long creeping; leaflets ovate-deltoid to lanceolate, 3.7–8.2 × 2.2–3.4 cm; raceme with 8–15 flowers, inner sepals reddish, ovate, 5–7 × 2–4 mm, 1/3 to 1/4 length of petals, petals yellow, horn shaped, 15–22 mm	***E.zhaotongense* G.W.Hu**
–	Rhizome compact; leaflets elliptic or broadly ovate, 3.2–9.5 × 2.5–6 cm; raceme with 11–20 flowers, inner sepals pale yellow, 6–7 × 3.2–3.7 mm, more than 1/2 length of petals, petals pale yellow, nearly straight, 7–12 mm	***E.enshiense* B.L.Guo**
37	Rhizome always compact; flower stem usually with 2 opposite leaves; inflorescences glabrous; flowers ca. 4.5 cm in diam.; inner sepals pale yellow, ca. 2/5 length of petals, petals sulphur-yellow, ca. 20 mm	***E.franchetii* Stearn**
–	Rhizome long-creeping, sometimes compact; flower stem with 2 opposite leaves or 1 leaf; inflorescences glandular; flowers 3–4 cm in diam.; inner sepals whitish, pale pink or rose, 2/3 to 3/4 length of petals, petals pale purplish red, rose, pale yellow or whitish, 13–26 mm	**38**
38	16–80 cm tall; inflorescences raceme, sometimes panicle, 8–24 (–36) flowers, ca. 3 cm in diam.; inner sepals narrowly ovate, 8–10 × 4–6 mm; petals horn-shaped, 13–20 mm	***E.glandulosopilosum* H.R.Liang**
–	12–50 cm tall; inflorescences always raceme, 3–14 flowers, ca. 3–4 cm in diam.; inner sepals narrowly lanceolate or narrow elliptic, 11–20 × 3–7 mm; petals horn-shaped, 15–26 mm	**39**
39	Rhizome always long-creeping, 1–3 mm in diam.; leaflets ovate or narrowly ovate, 3–11 × 2.3–5.7 mm, almost glabrous with only a few scattered hairs; inner sepals narrowly lanceolate, 15–17 × 3–4 mm; petals purplish red, 15–20 mm	***E.sutchuense* Franchet**
–	Rhizome long-creeping or compact, 1–5(–8) mm in diam.; leaflets narrowly ovate or ovate, 3–16 × 2–8.6 cm; inner sepals narrowly elliptic, 11–20 × 4–7 mm; petals rose, pale purple, occasionally yellowish, 15–26 mm	***E.leptorrhizum* Stearn**
40	Leaflets broadly ovate or ovate, 4–6 × 2–3 mm; flower stem with 2 opposite or alternate leaves; inner sepals red, 1/5 to 1/4 length of the petals	***E.membranceum* K.Mey.**
–	Leaflets narrowly lanceolate, lanceolate, narrowly ovate, or ovate; flower stem with 2 opposite or occasionally 3 whorled leaves; inner sepals white, pale yellow, purple or reddish-purple, 1/4 to 3/4 length of the petals	**41**
41	Leaflets abaxially densely and fully covered with long and tangled white hairs, thick as a blanket; inner sepals 3/4 length of the petals; petals deep purple; spur 2.3–3 cm	***E.zhushanense* K.F.Wu & S.X.Qian**
–	Leaflets abaxially glabrous or pubescent; inner sepals 1/4 to 2/3 length of the petals; petals white, pale yellow, yellow or purple; spur13–25 mm	**42**
42	Flower purple, purplish or yellowish; pedicels glabrous or occasionally glandular, 1–5 cm; inner sepals 1/2 to 2/3 length of the petals	**43**
–	Flower always yellow; pedicels glandular, 1.5–2 cm; inner sepals 1/4 to 1/3 length of the petals	**44**
43	40–60 cm tall; rhizome always compact; leaflets thick, fleshy, ovate to narrowly ovate, 4.1–5.6 × 2.1–2.7 mm, abaxially glabrous; flower 2–4 cm in diam.; inner sepals margin corrugated, ovate-elliptic, 6–21 × 3–9 mm; petals a little longer than inner sepals, 1.3–2.0 cm; chromatids 4n=24	***E.yingjiangense* M.Y.Sheng & X.J.Tian**
–	20–80 cm tall; rhizome compact, sometimes long creeping; leaflets leathery, narrowly ovate to lanceolate, 3–19.8 × 1.5–8.9 mm, abaxially with dense or sparse hairs or glabrous; flower 3–5 cm in diam.; inner sepals with smooth margin, ovate-elliptic, 6–21 × 3–9 mm; petals much longer than inner sepals, 15–25 mm; chromatids 2n=12	***E.acuminatum* Franch.**
44	40–86 cm (usually 70–80 cm) tall; leaflet lanceolate to narrowly lanceolate, 11.5–17.6 × 2.7–4.1 cm, abaxially glabrous or sparingly pubescent; inflorescence panicle, 20–100-flowered, 20–40 cm long, glandular; inner sepals ovate, 5–6 × 3 mm, inner sepals 1/4 length of the petals; spur horn-shaped, 15–25 mm	***E.jingchengshanense* Y.J.Zhang & J.Q.Li**
–	28–59 cm tall; leaflet lanceolate, 8–12 × 1.2–4.1 cm, abaxially sparingly or densely pubescent; inflorescence panicle, 25–32-flowered, 12–23 cm long, glandular; inner sepals elliptic or narrowly ovate, 10–12 × 5-6 mm, inner sepals 1/2 to 1/3 length of the petals; spur horn-shaped, 20 mm	***E.ilicifolium* Stearn**

## Discussion

Although the genus *Epimedium* is colourful in flower, from white, through yellow, to rose and purple, intraspecific flower colour variation is relatively uncommon. Before this study, only the polymorphism of *E.grandiflorum* and *E.acuminatum* has been described in the monograph or in the *Flora of China* (Stearn 2002; Ying et al. 2011). In addition, the colour variation of *E.glandulosopilosum* (yellow or pale purple) (Zhang et al. 2011) and *E.pseudowushanense* B.L.Guo (pale purple rose or purple, occasionally pale yellow) (Guo et al. 2007) have been mentioned. Since 2012, systematic studies and illustrations on morphology variation of *Epimedium* have been conducted by our group and we found five species with abundant intraspecific variations in flower colour.

*Epimediumacuminatum* was the species with the best-known colour variation. The flower colour of *E.acuminatum* was described as “white, yellow, rose-purple or pale violet” in *Flora of China* (Ying et al. 2011). And variations ranging from whitish, pale yellow, yellow, pale purple, purple-whitish to purple or reddish-purple have been noted in the specimens. Additionally, two species, *E.simplicifolium* and *E.chlorandrum*, akin to *E.acuminatum* have been described. The flower colour of *E.simplicifolium* was originally described as yellow (Ying 1975). It was subsequently revised to be purple or yellow (Ying 2001), and then revised again to reddish purple by the same author (Ying et al. 2011). *Epimediumchlorandrum* is notable for its anthers being green and the flowers being very pale primrose yellow (Stearn 1997). But yellow flower and polymorphism from yellow to pale reddish-purple have been described in its specimens *B. L. Guo 0606* and *B. L. Guo 0608*, respectively. In 2015, Zhang et al. found that the diagnostic characters of *E.simplicifolium* (with unifoliolate leaves) and *E.chlorandrum* (with green anthers and pollen) were within the range of morphological variations of *E.acuminatum* and finally were treated as synonyms of *E.acuminatum* (Zhang et al. 2011, 2015). Now, all of these descriptions and revisions can be well explained. *Epimediumacuminatum* is one of the most widespread species in the genus, and exhibits much variation in flower colour and other morphological characters (Zhang et al. 2015). And the polymorphism of flower colour exists both among populations and among individuals within populations.

It is very interesting and unexpected that we observed abundant intraspecific flower colour diversity. Our study suggests that the flower colour is not as stable as previously imagined. Therefore, flower colour seems to be a feature liable to great variation within some species and its taxonomic value should be discounted. From the geographical distribution of the five species, the results are consistent with the viewpoint that the comparatively unstable species often occur in western China where the genus is best represented and its evolution is still ongoing (Stearn 2002).

Although abundant flower colour variation has been observed in the present study, the reason for, or mechanism of, the colour variation is still unclear. The natural variation in flower colour may occur via the deposition of various anthocyanin pigments (Yang et al. 2010). Substantial variation in flower colour can be due to the differences in the presence, amount, or type of the carotenoid pigments (Wessinger 2015). In general, flower colour polymorphisms appeared to be a natural starting point. Flower colour polymorphisms are widely used as model traits from genetics to ecology (Alan 2007; Takahashi et al. 2013; Wang et al. 2017; Vaidya et al. 2018). In the present study, geographic variation in flower colour pattern within *E.acuminatum* showed a north-south geographic trend. The specimens with yellow flowers are mainly from the northern region of its distribution, while those with purple flowers are usually from the southern. The specimens with mixed colours occurred in the northwest of its distribution area (Fig. 2). The geographic pattern suggests that the variation in colour may be influenced by climate and ecology. Additionally, variation in flower colour has commonly been interpreted as adaptive. The differentiation in flower colour therefore was considered an important factor in promoting plant speciation (Bradshaw et al. 1995; Matsumura et al. 2006; Hopkins and Rausher 2011) or promoting adaptive, resulting from the disruptive selection by different pollinators (Mascó et al. 2004; Irwin and Strauss 2005; Veiga et al. 2015). On the other hand, the reproductive system may influence the patterns of variation in some taxa, and might account for the morphological complexity. Some colours, especially the continuous colour variation or the transition colours, may be generated by hybridisation, including hybrid speciation, historical hybridisation and ongoing speciation. Strong evidence for an outbreeding system and no internal barrier to hybridisation (high incompatibility and cross-ability) in *Epimedium* species has been proved (Suzuki 1983, 1984; Sheng et al. 2011). *Epimedium* are promiscuous, with bees creating garden hybrids and natural hybrids in the wild (Stearn 2002; Avent 2010; Horie et al. 2012). So far, more than 20 hybrids have been found in field or in garden cultivation (Stearn 2002; Avent 2010). For example, in the wild, on Emei Mountains, *E.acuminatum* was hybridised with *E.fangii* and produced a hybrid swarm (named *E.×omeiense*) (Stearn 1995). Hybrids tend to have different colours which enriches the colour variation (Stearn 2002; Avent 2010; Horie et al. 2012). Hybridisation may be one of the mechanisms of colour variation in *Epimedium*.

From another perspective, our results could have important implications for the utilisation of germplasm. Bearing lovely foliage and graceful flowers, *Epimedium* plants were previously mainly introduced as garden plants in Europe and America (Stearn 2002; Ward 2004). The species from China and their hybrids are about to set the gardening world on fire (Probst 1998). With great commercial prospects, *Epimedium* had received increased attention from cultivators. The abundant flower colour variations of intraspecific are of great significance in promoting their ornamental value and in creating many possibilities (Avent 2010).

## Conclusion

In this study, based on the extensive field investigation of populations during flowering seasons, comprehensive descriptions and illustrations for five *Epimedium* species were established. The flower colour used to be an important character in delimiting species in *Epimedium* (Stearn, 2002; Ying et al. 2011), but according to our results, flower colour might be too variable in the genus to be used in species delimitation. The flower colour and other characteristic variations in *Epimedium* are more extensive and complex than previously recognised. Therefore, it is not surprising that the *Epimedium* is taxonomically difficult and bewildering. The present study suggests that the flower colour is not constant at least in some species of *Epimedium*, which could be important to further taxonomic and evolutionary study.

## Supplementary Material

XML Treatment for Epimedium acuminatum

XML Treatment for Epimedium leptorrhizum

XML Treatment for Epimedium pauciflorum

XML Treatment for Epimedium mikinorii

XML Treatment for Epimedium glandulosopilosum
